# Therapeutic recreation camps for youth with childhood-onset systemic lupus erythematosus: perceived psychosocial benefits

**DOI:** 10.1186/s12969-022-00702-w

**Published:** 2022-06-07

**Authors:** Cristina Saez, Lorien Nassi, Tracey Wright, Una E. Makris, Justin Kramer, Bonnie L. Bermas, E. Blair Solow, Nicole Bitencourt

**Affiliations:** 1 University of Texas Southwestern Medical Center, Dallas, Texas USA; 2 Texas Scottish Rite Hospital for Children, Dallas, Texas USA; 3 Veterans Administration North Texas Health Care System, Dallas, Texas USA; 4 Texas A&M University, College Station, Texas USA; 5 Loma Linda University Medical Center, 11175 Campus Street, Coleman Pavilion, Loma Linda, CA 92354 USA

**Keywords:** Psychosocial health, Lupus camps, Transition to adult care

## Abstract

**Background:**

The psychosocial burden of having a chronic disease can be substantial for adolescents with childhood-onset systemic lupus erythematosus (cSLE). Current literature is scarce on interventions that can improve psychosocial outcomes for this population. Therapeutic recreation camps have been proposed as a beneficial experience for chronically ill pediatric populations. However, their effective components have not been well characterized in patients with cSLE. In this study, we sought to understand the various components of the camp experience for adolescents with cSLE from both the patient and parent perspective.

**Methods:**

We recruited patients with cSLE who had participated in one or more annual, weekend-long recreational lupus camp(s) near Dallas, Texas. Semi-structured in-depth telephone interviews were conducted from March-June 2020 with both the patients and parents. Questions focused on overall patient experience, psychosocial impact of camp participation, coping skills gained, and opportunities to prepare for the transition from pediatric to adult care. Interviews were coded and analyzed using inductive thematic analysis.

**Results:**

We interviewed 9 current and former campers (ages 16–24), including a current camp counselor, and 3 of their parents separately. Reported benefits included a positive impact on social support through peer bonding, opportunities to develop coping mechanisms through structured activities and peer/medical staff interactions, opportunities for education about the cSLE disease experience, improved adherence through peer modeling, overall increase in self-efficacy, and better parental insight into the patient experience. Participants also provided suggestions for expansion and improvement in program development to optimize educational opportunities for both campers and parents. In addition, they advocated for longitudinal social support and community building.

**Conclusions:**

In this qualitative study, in which cSLE patients and their parents reflected on their experiences with therapeutic recreation camps, we found several perceived benefits impacting the patient and parent experience. Participants expressed a desire for more educational opportunities that could contribute to their successful transition from pediatric to adult care. Further studies are needed to demonstrate the effects of therapeutic recreation camps on the psychosocial health of this population.

**Supplementary Information:**

The online version contains supplementary material available at 10.1186/s12969-022-00702-w.

## Introduction

Childhood-onset systemic lupus erythematosus (cSLE) is a complex, often aggressive disease [1] with implications for the psychosocial health and well-being of young people [2]. Health-related quality of life has been shown to be lower among youth with cSLE, especially among those with active disease and disease-related damage [3–5]. Few studies report on interventions with potential to improve quality-of-life outcomes in this population [6].

Therapeutic recreation camps have been proposed to enhance quality of life among youth with chronic illnesses [7]. There is evidence suggesting short-term psychosocial benefits of camps for children with chronic diseases such as diabetes, cancer, juvenile arthritis, and inflammatory bowel disease, including improvements in social interaction, acceptance, self-perception, and autonomy [7, 8].

To our knowledge, no research has focused on camps for youth with cSLE and their potential impact on the psychosocial health of these individuals. We report on the qualitative experiences and perceived benefits of lupus camps among a cohort of adolescents with cSLE, their parents, camp counselors, and former camp attendees who have now transitioned to adult care.

## Methods

### Setting and sample

We recruited a sample of persons who had previously attended lupus camp and their parents, including current pediatric campers (*n* = 5), former campers who have transitioned to adult care (*n* = 4), including one who served as a counselor, and the parents of current campers or counselors who have transitioned to adult care (*n* = 3). Participants were identified by reviewing a list of campers or counselors over the previous four years. Families with upcoming appointments who were felt appropriate for an interview were approached for consent. Lupus camp occurs yearly over a three-day weekend, during which 15 to 20 campers between the ages of 10 to 17 engage in both traditional camp activities (such as zip-lining, wall-climbing, crafts, fishing, etc.) and in an educational ‘ask-the-doc’ session with a pediatric rheumatologist. Campers also participate in various activities within their cabins, which are separated by age and gender and contain two camp counselors per cabin. Counselors are often former campers, Child Life staff (in-hospital professionals trained in helping children cope with stressful situations), or others with significant involvement in the lupus community.

After being informed of the study by their current or former physician, prospective participants were contacted by mail and then by phone to assess interest in participation. Participants were provided a modest compensation for their time ($30 debit card, which was reduced to $22.40 if they preferred not to disclose their social security number, in accordance with United States tax law). Institutional Review Board approval was obtained from the University of Texas Southwestern Medical Center (UTSW) and site approval granted from Children’s Health. Verbal informed consent was obtained from all study participants as approved by the IRB; all interviews occurred virtually in the early weeks of the COVID-19 pandemic.

### Data collection

Semi-structured in-depth interviews (*n* = 12) were conducted between March and June 2020 via telephone (per COVID-19 restrictions). A discussion guide was developed to query participants on their experience at lupus camp, skills learned, and how attending camp affected the psychosocial aspects of coping with cSLE (Additional file 1: Appendix A). A subset of questions was also geared towards an expected or realized transition from pediatric to adult care, which is addressed in a separate manuscript. The Qualitative Research Committee (QRC) at UTSW collaborated in this process, reviewing the guide for clarity and comprehensibility.

Interviews were conducted by a member of the QRC (JK) with formal training in qualitative research and facilitating one to one semi-structured interviews. Recruitment ceased among campers when thematic saturation was achieved, with little to no new concepts emerging through continued interviews [9]. All interviews were digitally audio-recorded and transcribed verbatim by a professional transcription service.

### Data analysis

An inductive thematic analysis approach was used to identify emergent themes within the interview data. Transcripts were independently coded and analyzed using constant comparative analysis by two members of the QRC to identify salient themes and reconcile any coding discrepancies. They collaboratively developed and refined a coding dictionary throughout data analysis. NVivo 12 Plus software was used to manage findings during data analysis.

## Results

Fifteen people were invited to participate, all of whom agreed and provided verbal consent. Twelve were reachable via telephone and available for interview. We conducted twelve in-depth interviews averaging 43 min (range 25 to 66 min). Patients and parents were interviewed separately. Participants had the following demographic breakdown: all women, 8 Non-Hispanic Black, 3 Hispanic of any race, and 1 Non-Hispanic Asian. Participants were roughly representative of our patient population with cSLE (which is predominantly female (86%), 43% Hispanic (of any race), 35% non-Hispanic Black, 15% non-Hispanic White, 7% non-Hispanic Asian, and mostly publicly insured (61%)). Pediatric campers ranged from 16–18 years of age, while former campers (now in adult care) ranged from 20–24 years of age. Most campers had participated in lupus camp more than once and the gap between camp participation and interviews ranged from six months to three years.

We identified several themes surrounding attendance at lupus camp, including social support, effects of lupus camp on coping, opportunities for learning and education, adherence and navigating care, insights into the patient experience, and suggestions for expansion and improvement.

### Social support

Social support emerged as an important theme and a leading reason for attending lupus camp (Table 1). Shared experiences among campers and between campers and counselors provided a basis for social connectedness. One parent found that after attending lupus camp, her daughter “had a better understanding that she was not by herself, and looked forward to going back [and sharing] her story with everybody else.” Participants recounted an inclusive environment where “[we are not] criticized for being open,” and other campers represented “the people who will most understand you.” Attending camp was described as a safe outlet for feelings and a way to normalize the experience with lupus: “[we both went through] something that was so traumatizing [lupus flare]…but they’re recovering [too]…I’m not alone.” Several participants spoke to how social support and sharing their experiences allowed them to cope with lupus-related difficulties and empowered them to persevere. There was a general understanding that “[the other campers] got your back and everything will be okay.” One counselor was encouraged to see campers being “proactive, taking initiative [to inspire] each other on their own.” Ongoing engagement with other campers after the conclusion of camp through phone or social media allowed some participants to consider they “have connections from camp to rely on.”

**Table 1:** Social Support

**Shared experiences** They got to share their stories and that really helped her to come to grips with I’m not by myself, somebody else is experiencing arthritis, somebody else is experiencing the rashes… (parent)
**Bonding, understood** …they all understand [and] can sit around and talk to each other about their insecurities and how they feel and no one judges them …finally I have an outlet where I can talk to someone that knows exactly how I feel and I don’t have to constantly explain… (parent)
**Social support** …it’s very positive and fun and exciting … it’s just real life, and they’re just letting you know that- hey, you’re not in this alone. There’s other people out there and they can actually help you get through a hard time in your life because it’s very hard knowing that you have an incurable disease and how to deal with it…[and others can] give you tips and pointers and ideas on how to maybe change some things up… (parent)
**Normalizing**: [Lupus camp] was like a breath of fresh air. I felt normal to be with other people going through the same thing I go through. (patient)
**Empowered** They’re gonna make new friends, sometimes life-long friends…You get to be in an environment where there are other people that are experiencing the same things that you are and it could be motivating for you to live your life to the fullest without being hindered by lupus or having the thought of being held back because of the lupus. (parent)
**Ongoing engagement** …personally it did help me having other people with the same illness … being able to compare the things we went through and just not feeling alone with the illness [because] it feels like really alone. So it was nice to have other people I could talk to and even exchanging numbers and stuff with some of them so I could keep in touch. (patient)

### Effect of lupus camp on coping

Participants spoke to the respite that lupus camp provided, such that, paradoxically “[at camp] I don’t have to always think about lupus.” Campers are afforded the opportunity to enjoy the weekend and build their coping resources (Table 2). Participants recounted greater acceptance of their disease, enhanced confidence to do ‘normal’ things, and tangible actions they learned to help manage stress or improve their sense of control over their illness. Parents noted an increase in self-efficacy and felt their children had “a little more confidence about themselves” and began to learn “to stand up or speak up for [themselves].” Several participants correlated overcoming the fun challenges at camp (such as obstacle courses or rock climbing) with a metaphor for life and felt these gave them a different mindset when returning home. Participants were grateful for the opportunity to explore their identity outside of lupus.

**Table 2:** Effect on Coping

**Control over identity** …to be who you are, and own lupus, don’t let it own you…[learned from] the campers [who had lupus for] longer being their own person, [and continue to be] who they are… (patient)
**Acceptance** [Lupus camp] got me out of my shell because I knew people were like me around me, so it helped me with my accepting having lupus. (patient)
**Cognitive reframing of limits** They would always have us doing a lot of active work like [rock climbing and even zipline], and it helped in a way… we might be considered weaker because of our immune system, but [we don’t] have to let that take over us… it helped me get in the mindset of- oh, I might feel like I’m a lot weaker, but I can still do all of this stuff. (patient)
**Action over Fear** I know how to take control of [my health]. I can’t be scared of the sun all the time… I love that [we teach campers to use] sunscreen to protect yourself [and] not be scared. (patient)
**Positive outlook** …after the first camp, she had positive outlook on things and she was able to hear others’ experience with it [and] become more aware of what was possible …and that [lupus] could be managed and that she could have a full life not hindered by her lupus… (parent)
**Courage, metaphor for life** …it’s more of a mindset thing, but what excites me about the obstacle course is that it puts a lot of pressure on your body… and it’s a motivator because it’s really an accomplished feeling when you’re at the top … I always feel a sense of determination- I have to do it. I have to show the younger kids that I’m not scared, though I am definitely terrified. (patient)
**Confidence, learned skills** …she got more confident [after coming back from lupus camp]. She was able to take someone else’s experiences [and] how they handle certain situations and …do some of that in her life and make some changes, and she benefited from it. (parent)

### Opportunity for learning, education

Camp provided educational opportunities on various aspects of living with lupus (Table 3). Informal conversations among campers and counselors allowed for learning from shared experiences and complimented a more formal educational “ask-the-doc” session. Participants “especially liked the …ask the doctor sessions [where] anonymous [questions could be asked] and answered” in a more comfortable, intimate space for group dialogue. Through different modalities, participants felt more informed about lupus symptoms, healthy eating, medications and their side effects, and the importance of medication adherence. Transition-related topics were also addressed and camp counselors, who were previous campers, provided insights into their experiences in adult care.

**Table 3:** Opportunity for Learning, Education

**Learning about lupus** …now I know more …I understand a little bit more about [lupus]. (patient)
**Symptom management from others with lupus** The counselors [told their story] and … a lot of us all shared the same symptoms… they taught me how they deal with [lupus], and sometimes I utilize what they taught me, and it helps me. (patient)
**Education on medication** It was nice at least hearing more about it [prednisone] because I had never really known much about the medication I was taking. (patient)
**Sharing pointers on medication adherence** …so I explained [to fellow campers that] when I first started taking medication, I was really bad at swallowing [because] the taste of the medication would stay in my mouth, so I mentioned how I would always eat a lollipop or something right after I took a pill so I didn’t have to deal with the taste in my mouth. (patient)
**Preparation for transition** With me getting ready to leave [pediatric care] they were giving us advice on how to be prepared…[including advice about] insurance and what can we do to stop [flare ups]…[I talked] to counselors and nurses and doctors while at camp [about] what to expect and …it was helpful. (patient)
**Sharing advice** She couldn't wait to get back out there now on the opposite side [i.e. as an adult]—she enjoyed helping someone else know how she got through the whole process. (parent)
**Opportunity for more frank education** When they’re all sitting together, the doctors could emphasize how important it is for them to take and know their medication and the side effects … And it would give the kids an opportunity to say- what if I stop taking mycophenolate? And I’d like for it to be in a safe place, a place of comfort where they can feel free to ask that question and get an *answer* answer, not just something blown off. (parent)

### Navigating health care and modeling adherence

Campers and their parents discussed how skills learned at camp impacted them (Table 4). Improved medication adherence was one potential benefit as described by a parent: “she was very proactive in taking her medicines [after going to lupus camp and was] also aware of the timings that she would take her medicine.” Campers reported sharing tips for pill-taking and advice for staying organized. Modeling of medication adherence by other campers and by counselors occurred, as did interactions between campers and the healthcare team, which was reported to facilitate communication and foster greater collaboration in clinical settings.

**Table 4:** Navigating Health Care, Modeling Adherence

**Skills learned** [Skills learned at camp include] taking her medicine timely, setting reminders of taking her medicine, writing down new things that occur so the next time she visits the doctor she will bring that up, and just taking cues from her body…[skills learned] mainly [from] her peers, but also her doctor. (parent)
**Increased responsibility** [Attending camp] had made her a little bit more responsible. [She now] actually answers the phone and sets up her own appointments. (parent)
**Communicating with physicians** Having a doctor comfortable [with] questions was nice because it made me aware that it is okay to bring them up or ask my doctor… I had never really known much about the medication I was taking [until then]. (patient)
**Self-efficacy, independence** [Attending camp] give[s] her the confidence to be able to communicate things that she’s learned about herself, her body, the condition, what works for her, what doesn’t work, and she’s able [to say] I think we need another approach…speaking on her own behalf. (parent)
**Increased control of health** I eventually had to tell [my parents] this is my health, I know what is best for my health, and I know how to take control of it. (patient)

### Insights into the patient experience and suggestions for expansion and improvement

Parents felt they gained valuable insights into their children’s illness experience through camp-mediated conversations (Table 5). While all those interviewed spoke highly of the camp experience, patients and parents also cited opportunities for further program development (Table 6). Parents welcomed educational sessions targeted at helping them understand lupus and sharing strategies with other parents about how to cope and help their children cope with the challenges of managing SLE. Campers and counselors were interested in an organized method of ongoing engagement with other participants throughout the year. They expressed interest in hearing from physicians of other specialties and adults living with SLE, as well as having a structured platform for continuing social support. One cited ‘big brother/big sister’ type programs as a potential model to provide continued support and expressed a desire for camps aimed specifically at transition-aged persons. Participants also suggested that community organizations such as the Lupus Foundation were well positioned to organize events aimed at patients and their families.

**Table 5:** Insights into the Patient Experience

**Parental insight into child’s experience** It’s good for a parent to go…to see my child be a lot more comfortable and open and you find out their insecurities or whatever bothers them that you didn’t know because they won't really tell you and you find out just by listening in and you’re like- oh my gosh, [I] never [knew] that. So I think that every parent should take the opportunity to go with their kid. (parent)
**Opportunity for friends to learn about lupus** I’d strongly recommend [going to lupus camp]. I would talk about it really positively to my friends who didn’t fully understand my illness… they always seemed supportive [and] curious about my illness and [then] tried to understand it. (patient)
**Family understanding** …teach the family to understand them because sometimes they [are] really sick, and we try to see them as normal, but I we don’t know how they feel [even though] as family [we] try to understand them. (parent)

**Table 6:** Suggestions by Patients and Parents

**Camp for transition-aged youth** I really wish there was some type of transition camp for young adults [with lupus] transitioning into adult care… (parent)
**Events for parents** I would be the first one in the door [for an event for parents] because I would like to know how to handle certain situations, especially when this transition happens. (parent)
**Information for parents** …the information that I received [as a parent] was so broad and so frightening [it was] overwhelming…more education for families [would be nice]… just helping them to realize there are children [and] other parents experiencing the same thing…what did you do to cope with this and help your child cope, what did work, what didn’t work… (parent)
**Shorter meetings between camps** …other camp-type [events] could be sponsored throughout the year … bringing in other doctors from other areas… like guest speakers or specialists not just about adult healthcare [but also] just handling lupus from a different perspective. (patient)
**Keeping in contact** There should be little texts sent out or even little Zoom meetings for us to keep in contact with each other and the doctors could see us throughout the year. (patient)
**On hypothetical meetings occurring between camps** …at the beginning of the meetings new people [would] hear from the doctors and the more experienced lupus patients or bring in people [with] lupus and [explore] career [and family] goals and show us we can do things… (patient)
**Big brother, big sister** …maybe a big brother, big sister program …where the older lupus kids can sponsor a younger one throughout the year… (parent)
**Engaging community organizations** Maybe [there can be] more involvement [of organizations like the] Lupus Foundation… because I personally am passionate about lupus and helping others who are struggling with it, [who] went through what I went through. (patient)

## Discussion

In this study we describe the experience of current and former campers and several parents with a therapeutic recreation camp for children and adolescents with cSLE. The perceived benefit to attending lupus camp occurred in several domains, including improved social support and peer bonding, an opportunity to build coping resources, to interact with medical professionals and to observe medication adherence. Participants reported improved knowledge surrounding cSLE, and parents felt they gained insight into their child’s illness experience. Suggestions for expansion and improvement were described.

Our finding that lupus camp provides opportunities for social support and improved disease knowledge is consistent with the available literature on therapeutic recreation camps. One study of a residential summer camp for children with malignancies confirmed the highly supportive environment of camp, with participants reporting more emotional/informational support/esteem-enhancing support, and tangible support compared to the general population [10]. Connectedness with peers has been shown to mediate the relationship between psychological adjustment and optimism among children with chronic illnesses [11]. The opportunity to build coping skills was highlighted by participants of our study and is a theme echoed by others. Residential camps for children with diabetes identified several psychosocial benefits, including a decrease in distress and improved independent self-care [12], self-efficacy [13], self-perception, and resilience [8].

Attending therapeutic recreation camps has been associated with better health-related quality of life [8] and disease outcomes [14], though the specific underlying reasons for these outcomes have not been fully elucidated. Our participants suggested that camp provides an opportunity for participants to observe and model adherence and to interact with medical staff, which may facilitate optimal healthcare interactions outside of the camp setting.

Adults with childhood-onset SLE are at risk for long-term adverse consequences [15], and the transition from pediatric to adult care is a particular vulnerable period for emerging adults [16]. Complex interventions are needed to address the needs of this high-risk population, and camp may provide an opportunity to enhance transition-related skills. Multicomponent interventions have been successfully introduced through a camp-based program among children with other chronic illnesses, including those with type 1 diabetes, with subsequent improvement in glycemic control and parental stress [14]. Whether similar strategies could improve cSLE transition outcomes deserves further study.

Limitations of this study include that it was conducted among a small number of former and current campers and their parents from one major metropolitan area. Findings reflect their experience with one specific lupus camp, and generalizability is thus limited. The experience of participants in this study may not reflect all campers’ experience and is by necessity subjective. Those who attend lupus camp may also be fundamentally different from those who elect not to participate or who are not eligible to participate due to the severity and unstable nature of their illness. Additionally, recruitment was limited by financial constraints. Interviews occurred during the beginning of the COVID-19 pandemic, and how the decreased ability to socialize with peers influenced recall and responses is unknown. Qualitative studies are meant to be hypothesis generating; further research is needed to corroborate these preliminary findings, including among participants from other lupus camps. Future work should aim to quantify improvements in psychosocial benefits including validated questionnaires that measure changes in independent self-care, self-efficacy, resilience, and health-related quality of life [8, 12, 13].

## Conclusion

Several important implications can be drawn from this work. Recreational lupus camps may be an effective avenue to improve the psychosocial well-being of children and adolescents with cSLE, with the potential for improved social support, peer bonding, coping, self-efficacy, disease acceptance, and educational opportunities. The effect of camp on participants’ interactions with medical staff and the healthcare system requires deeper investigation, and how best to expand the camp experience and incorporate it into clinical care should be explored.

## Supplementary Information

**Additional file 1. **

## Data Availability

The datasets used and/or analyzed during the current study are available from the corresponding author on reasonable request.

## Abbreviations

cSLE: Childhood-onset systemic lupus erythematosus
UTSW: University of Texas Southwestern Medical Center
COVID-19: Coronavirus disease 2019
QRC: Qualitative Research Committee
JK: Justin Kramer (author)
SLE: Systemic lupus erythematosus
