# Supplementary material for: Therapeutic recreation camps for youth with childhood-onset systemic lupus erythematosus: perceived psychosocial benefits
Source: Pediatr Rheumatol Online J. 2022 Jun 7;20:39. doi: 10.1186/s12969-022-00702-w (PMC9171737; doi:10.1186/s12969-022-00702-w)
Supplement: Supplementary file 1 — Additional file 1. [file 12969_2022_702_MOESM1_ESM.docx]

**Overall Purpose:**

- Explore the role of lupus camps in providing patients and parents with skills to manage healthcare needs, develop resilience, and prepare for transition
- Discussions of patient and parent resilience in handling difficult situations and remaining engaged in care
- Understand patients’ and parents’ experiences with pediatric care as they approach transition to adult rheumatology

**Eligibility Criteria:**

- Current pediatric patients and their parents

**Patient Centered Questions**

**I. General Transition**

1. When you think about transitioning to an adult doctor, how does that make you feel? Why?
2. Are there things that you are looking forward to when it comes to transitioning to an adult doctor?
   1. Is there anything you’re concerned about?
3. Have you had conversations with your pediatric doctors about going to see an adult doctor?
   1. How did those conversations go? Were they helpful?
   2. Did you speak with anyone else on the medical team about transitioning? Were they helpful?
4. Are there specific things that your pediatric doctors, nurses, or any other members of the medical team (e.g. social worker) can do to help you in the process of transitioning to an adult doctor?
5. What role do you think your parents (or guardians) should play in the process of transitioning from a children’s doctor to an adult doctor?

**II. Lupus Camp(s)**

1. Have you ever attended lupus camp? If so, can you tell me a bit about that experience?
   1. What were some of the things that you liked?
   2. What were some of the things that you think could’ve been improved?
2. Did you find that meeting other young people with lupus, either the campers or counselors, helped you in any way? How?
3. Were there things that you learned from the people you met at lupus camp? Like what?
4. Did attending lupus camp teach you anything about lupus or the medical care system?
   1. Can you give me an example? Was this helpful?
   2. Who were the main people who helped you learn these things?
5. Did seeing other young people who were prescribed lupus medications affect you?
   1. Did you ever talk with them about taking medications? For example, strategies to make sure you don’t forget or just some of the struggles with all the meds?
6. Did going to lupus camp help you in learning how to live with lupus?
   1. This can be anything, such as changing your mindset regarding the disease, specific strategies to manage flares, or even ways to cope with medications.
7. Are there things that could be done throughout the year to help remind you of what you learned at lupus camp? For instance, online meetings, in-person sessions, social media connections etc.
8. Would you recommend other youth with lupus go to lupus camp? Why?

**III. Lupus Camp and Resilience**

1. Do you think attending camp will have any impact on the transition process for you? If so, in what ways?
2. Are there things you learned at lupus camp that you use in your regular life now? This can be a skill that helps you take your medication, strategies for talking to doctors, or things that help you cope with your disease.
3. Have you ever had to deal with difficult experiences regarding your healthcare in the past, such as medical expenses or getting to appointments? How did you deal with these situations?

**IV. Education**

1. How comfortable do you feel with your knowledge of lupus and of your medications?
2. Do you feel that you know what to do if you were having a healthcare emergency, like a flare-up?
3. When you have questions about lupus or your medications, where do you look for information?
4. What’s the best way for you to learn about lupus besides from your doctor? For example, handouts, websites, social media, youtube, friends, etc.

**V. Family and Friends**

1. How have your family or your friends helped you in terms of taking care of yourself?
   1. Are there any specific things that you rely on them for? What about with respect to healthcare?
   2. Are there things they could do to be more supportive?
2. Is there anything else that you want to say about lupus camps, your upcoming transition to adult care, or your healthcare in general?

**Parent Centered Questions**

**I. General Transition**

1. When you think about your teenager transitioning to an adult doctor, how does that make you feel? Why?
2. Have you had conversations with your teenager about what it will be like to go see an adult doctor in the future? Are there specific things that you bring up and what do those conversations look like?
3. Do you feel confident that your teenage child has the skills necessary to successfully make the switch to an adult doctor? For example, they know their medications, their disease, and can speak to the doctor themselves. Why?
   1. Are there any areas in which you think that they can, or need to, improve upon?
4. Are there things that you are looking forward to when it comes to your teenager transitioning to an adult doctor?
   1. Is there anything you’re concerned about?
5. Have you had any conversations with your child’s pediatric doctors about going to see an adult doctor?
   1. How did those conversations go? Were they helpful? Were there things that they emphasized?
   2. Did you speak with anyone else on the medical team about transition? Were they helpful?
6. Are there specific things that your teenage child’s pediatric doctors, nurses, or any other members of the medical teams (e.g. social worker) can do to help you and your child in the process of transitioning to an adult doctor?
7. What do you think is your role in the process of your teenager transitioning to see an adult doctor?

**II. Lupus Camp(s)**

1. Do you think going to lupus camp was beneficial for your teenager? How so?
   1. Did they mention anything specific about camp that they enjoyed or disliked?
2. Did you notice any changes in how your teen thinks about themselves or lupus after attending lupus camps? If so, what?
3. Did you notice any changes in taking medications, talking with doctors or nurses, or in learning how to live with lupus generally? Can you give an example or two?
4. Would you recommend other youth with lupus go to lupus camp and why?
5. What are things that could be done throughout the year to help remind your teen of the things they learned at camp? For instance, online meetings, in-person sessions, etc.
6. If available, would you want to participate in similar activities for parents of children with lupus?

**III. Lupus Camp and Resilience**

1. Do you think attending camp will have any impact on the transition process for your teenager? If so, in what ways?

1. Are there things your teen learned at camp that they use in their regular life now? This can be a skill that helps them remember to take their medication, talk with their doctors, or strategies to cope with their disease.
2. Have you or your child ever had to deal with difficult experiences regarding their healthcare in the past, such as medical expenses or struggles regularly attending appointments? How did you deal with these situations?

**IV. Education**

1. How comfortable do you feel with your own knowledge of lupus? How comfortable do you think your teenager is in their knowledge of lupus?
2. Do you feel that they know what to do if they were having a healthcare emergency, like a flare-up?
3. When you have questions about lupus or the medications that they are taking, where do you look for information? Where does your teen look?
4. What’s the best way for you and for your teen to learn about lupus besides from your doctor? For example, handouts, websites, social media, youtube, other parents etc.

**V. Family and Friends**

1. How have your family or your friends helped you while you learned to manage your child’s lupus?
   1. Are there any specific things that you rely on them for? What about with respect to your teen’s healthcare?
   2. Are there things they could do to be more supportive?
2. Is there anything else that you want to say about lupus camps, your teen’s upcoming transition to adult care, or their healthcare in general?
